# Interoception in Anorexia Nervosa: Exploring Associations With Alexithymia and Autistic Traits

**DOI:** 10.3389/fpsyt.2020.00064

**Published:** 2020-02-21

**Authors:** Emma Kinnaird, Catherine Stewart, Kate Tchanturia

**Affiliations:** ^1^ King’s College London, London, United Kingdom; ^2^ Maudsley Centre for Child and Adolescent Eating Disorders, South London and Maudsley NHS Foundation Trust, London, United Kingdom; ^3^ Eating Disorders, South London and Maudsley NHS Foundation Trust, London, United Kingdom; ^4^ Department of Psychology, Ilia State University, Tbsili, Georgia

**Keywords:** anorexia nervosa, eating disorders, autism, alexithymia, interoception

## Abstract

**Background:**

Previous research on whether interoception is altered in anorexia nervosa (AN) using the heartbeat tracking task has yielded inconsistent results. However, no previous research has examined whether interoception is associated with alexithymia and autistic traits in AN, conditions which are more prevalent in this population and thought to be related to performance in this task. The aim of this study was to explore whether altered interoception in AN is associated with alexithymia and autistic traits.

**Methods:**

We assessed interoceptive accuracy using the heartbeat tracking task in *n* = 37 people with AN, and *n* = 37 age and gender matched healthy controls (HC), and explored within the AN group if interoceptive accuracy was related to self-rated alexithymia or autistic traits. We also assessed self-reported interoceptive ability, and the relationship between subjective and actual performance.

**Results:**

Heartbeat tracking task performance was not found to be altered in the AN group compared to the HC group. However, confidence ratings in task performance in the AN group were lower compared to the HC group. Unlike the HC group, confidence ratings in the AN group did not correlate with task performance. Within the AN group there was no relationship between interoceptive accuracy, alexithymia, and autistic traits, after controlling for the potential confounders of anxiety and depression. There was a relationship between confidence ratings and illness severity in the AN group.

**Conclusion:**

The results found no differences between heartbeat tracking task performance in people with AN compared to HC. There was no association between task performance, alexithymia and autistic traits in AN. Results do suggest that people with AN exhibit lowered confidence in their task performance, and that they may lack insight into this performance compared to HC. The findings are discussed in the context of potential significant limitations of the heartbeat tracking task, with recommendations for future research into interoception in AN.

## Introduction

Anorexia nervosa (AN) is an eating disorder (ED) characterized by the restriction of energy intake resulting in low body weight, a resistance to weight gain, and altered body image (1). Early research on AN suggested that this food restriction, and associated symptoms such as altered body image and problems identifying emotions, may be driven by a difficulty detecting internal bodily sensations (2). This concept of sensitivity to bodily stimuli has come to be understood under the wider term of interoception, or “the sense of the physiological condition of the entire body” (3). Interoception encompasses how the brain identifies, interprets and integrates internal stimuli. Altered interoception is associated with a number of processes thought to be related to the development and maintenance of AN, including appetite regulation, emotion regulation, self-awareness, and motivation (3–6). Research into interoception has encompassed various definitions of key terms, including similar terms being used by different studies to describe different concepts (7). Recently, Garfinkel and co-authors (8) have defined interoceptive accuracy (objective ability to detect internal stimuli), interoceptive sensibility (self-perceived ability to detect internal stimuli), and an individual’s metacognitive insight into their objective ability. The current study will use these definitions when discussing different aspects of interoception, including when referring to previous research which used different terms.

Studies on whether interoceptive accuracy is altered in AN have yielded mixed findings. Although earlier research often focused on hunger and satiety detection, more recent studies on interoceptive accuracy in AN have most commonly used measures of cardiac interoception, specifically the heartbeat tracking task (9). Using the heartbeat tracking task, two initial studies found that people with AN had lower interoceptive accuracy (10, 11). By contrast, three more recent studies using the same measure found no significant differences between people with AN and healthy controls (HC) (12–14). One previous study has used a heartbeat discrimination task, finding no differences between people with AN and HC (15). By contrast, research on interoceptive sensibility in AN consistently suggests that people with AN self-report a lack of confidence in their ability to detect their internal stimuli compared to HC (16). It should be noted that these previous interoceptive sensibility studies have primarily used the interoceptive subscale of the Eating Disorder Inventory (EDI) (17). This subscale has been criticized for potentially measuring emotional, rather than somatic awareness (15), for not distinguishing between a lack of acceptance of emotional arousal, and a lack of clarity surrounding internal stimuli (18). The subscale also primarily focuses on the sensations of hunger and satiety, rather than including a range of different body sensations (13).

Therefore, previous research suggests that while people with AN self-report a lowered ability to detect internal stimuli, it is unclear whether this equates to objectively lowered interoceptive accuracy. One potential reason for this variability in previous findings is the methodology. The majority of studies on interoceptive accuracy in AN have used the heartbeat tracking task, but this method has come under increasing scrutiny. Heartbeat tracking can be influenced by a number of factors, including BMI (19), cardiac variables (20), and prior knowledge about typical heart rates (21). It has also been suggested that heartbeat tracking scores reflect participant beliefs about heart rate, rather than actual counted heartbeat sensations (22, 23). In addition, the test has low test-retest reliability, and does not relate to other measures of cardiac interoception (24, 25). An additional difficulty in using this test to measure interoceptive accuracy in AN is the potential influence of related clinical variables. For example, previous research has considered the role of depression and anxiety when exploring this area, variables known to be associated with altered interoceptive accuracy (11, 12, 26, 27). However, to date, no research has explored whether there is an association between alexithymia, autistic traits and interoception in AN.

Autism is a neurodevelopmental disorder associated with differences in social communication, and restricted behaviours and interests (1). People with AN exhibit heightened levels of autistic traits compared to HC (28), and qualitative research suggest that altered interoception could contribute to disordered eating in autistic adults (29, 30). Research suggests that interoceptive accuracy may be lowered in autism (31, 32), although other studies have found no differences in autism compared to HC (33–35).

It has been suggested that apparent differences in interoceptive accuracy in autism could in fact be related to the higher levels of alexithymia seen in autistic populations (36–40). Alexithymia is associated with lower interoceptive accuracy, to the extent that it has been hypothesised to be the product of impaired interoception (21, 41, 42). Furthermore, this relationship may be specific to clinical populations: a recent meta-analysis found no relationship between interoception in control populations, but found that lowered interoception was related to heightened alexithymia in EDs and autism (43). However, this study used a broad definition of interoception, described as “interoceptive awareness,” including attention, detection, magnitude, discrimination, accuracy, insight, sensibility, and self-report abilities surrounding bodily cues (7). Therefore, the findings related to a broadly defined construct of interoception, incorporating a number of different measurement approaches. Moreover, the meta-analysis considered EDs as a single category rather than distinguishing between AN, bulimia nervosa (BN) and binge eating. No previous study has specifically investigated the relationship between interoceptive accuracy as measured using the heartbeat tracking task and alexithymia in AN.

Therefore, any attempt to investigate the associations between autistic traits and interoception in AN would also require a consideration of the role of alexithymia, with research suggesting alexithymia is heightened in people with AN (44). However, to date the associations between different facets of interoception, alexithymia, and autism in AN have not been explored. The aim of this exploratory study was to address this gap in the literature by investigating the following hypotheses using the heartbeat tracking task:

1. People with AN would exhibit lowered interoceptive accuracy compared to HC.
2. People with AN would self-report lowered interoceptive sensibility compared to HC.
3. People with AN would exhibit poorer metacognitive insight into their task performance compared to HC.
4. There would be an association between interoceptive accuracy, alexithymia and autism within the AN group.

In the context of a lack of previous research in this area, the current study only examined associations between interoceptive accuracy, alexithymia, and autistic traits in AN. It does not present hypotheses surrounding the expected relationships.

## Methods

### Participants

Participants with AN (*n* = 37) were recruited from a specialist ED treatment service. Additional participants were recruited by advertising online with a UK-based ED charity. All participants met DSM-V criteria for AN as assessed using the Structured Clinical Interview for DSM [SCID-5 (45)]. Participants were excluded if they reported a neurological condition or serious medical condition. Participants with AN were included if they had a previous diagnosis of autism.

Age and gender matched HC (*n* = 37) were recruited through the local university and through advertising online. Exclusion criteria for HC included any history of EDs or mental health conditions, neurological or serious medical conditions, or a prior diagnosis of autism. These were confirmed through screening using the SCID-5 and the Autism Spectrum Quotient (46). Participants received £20 for taking part in the study.

### Measures and Procedure

#### Interoceptive Accuracy

Interoceptive accuracy was assessed using a heartbeat tracking task, which requires participants to detect their own heartbeats (9). Participants were asked to silently count their heartbeats during four randomised time windows (25, 35, 45, and 100 s), and then at the end of each window to report the number of counted heartbeats to the researcher. Participants were verbally cued to begin counting by the researcher, and then cued to stop counting when a timer alarm sounded. Participants then verbally reported the number of heartbeats counted. Actual number of heartbeats were measured using a pulse oximeter with the sensor attached to their index finger. An interoceptive accuracy score was calculated for each time trial for each participant using the formula 1 − (|nbeatsreal − nbeatsreported|)/((nbeatsreal + nbeatsreported)/2), with resulting scores averaged across the four trials to give an overall score for each participant (8).

Although the efficacy of the heartbeat tracking task as a measure of interoceptive accuracy has recently come under scrutiny, this task was chosen as it has been used in the vast majority of previous research on interoceptive accuracy in AN, alexithymia and autism (22, 47). As the aim of this study was to explore whether heartbeat tracking task performance could be related to alexithymia and autistic traits in AN, the current study has continued to use this method.

#### Interoceptive Sensibility

In the context of previous criticism of the EDI interoceptive subscale (13, 15, 18), interoceptive sensibility was assessed using total scores on the awareness subscale of the Porges Body Perception Questionnaire [BPQ (48)]. The subscale uses 45 questions to assess self-reported awareness of bodily symptoms, with participants answering on a Likert Scale from “never” to “always.” A higher score indicates higher interoceptive sensibility. The subscale has previously been used in interoception research, including in autistic populations, but has not previously been used in people with AN (8, 31). A recent meta-analysis found that the BPQ was significantly positively associated with alexithymia (43).

Interoceptive sensibility was additionally assessed using task confidence ratings: immediately following the heartbeat tracking task, participants were asked to rate how confident they were in their task performance on a scale from 1 (least confident) to 100 (most confident).

#### Metacognitive Insight

Metacognitive insight into performance was operationalized as the correspondence between interoceptive accuracy (heartbeat tracking task) and interoceptive sensibility [BPQ and confidence ratings (7, 49)]. In the present study this was measured as group correlations between heartbeat tracking scores, and BPQ and confidence ratings.

#### Clinical Variables

Alexithymia was measured using the Toronto Alexithymia Scale [TAS-20) (50)]. The TAS-20 is a self-report measure of alexithymia (the inability to label and describe emotions in the self) with good internal consistency and test-retest reliability. A higher score indicates higher levels of alexithymia. The TAS-20 is widely used in research in both autistic and ED populations (37, 44).

Autistic traits were measured using the Autism Spectrum Quotient [AQ) (6)]. The AQ is a continuous measure of autistic traits, with higher scores indicating higher levels of autistic traits. The AQ has previously been used in AN populations, with people with AN typically scoring higher compared to HC (28). Whilst the AQ does include a cut-off score, with scores above 32 indicating potentially clinically significant levels of autistic traits, recent research has questioned the ability of the AQ to distinguish “true” autism cases in populations with high levels of autistic traits (51–53). Consequently, beyond screening HC for high autistic traits at the beginning of the study, the AQ was only used in the analysis as a continuous measure.

Previous research has suggested that the relationship between alexithymia, autism and interoceptive accuracy cannot be successfully measured without accounting for the role of anxiety and depression (47). Therefore, anxiety and depression were measured using the Hospital Anxiety and Depression Scale [HADS (54)]. The HADS is a widely used 14-item self-rating instrument for anxiety and depression. The clinical threshold is 10 for each scale.

### Procedure

The study received ethical approval from North East - Newcastle & North Tyneside 2 Research Ethics Committee (18/NE/0193). All subjects gave written informed consent in accordance with the Declaration of Helsinki. All testing took place during a single study visit. Following informed consent, participants completed questionnaires and self-reported demographic information. Height and weight were measured on the day of testing to assess BMI scores. If a participant with AN was currently in treatment, their BMI was taken from their most recent measurements in clinical notes. Participants with AN additionally self-reported their illness duration. Participants then completed the heartbeat tracking task, and rated their confidence in their task performance. A small number of participants did not complete all questionnaires but did complete all screening measures and experimental tasks: any difference in group numbers across each self-report measure has been highlighted in the results.

### Analysis

Statistical analyses were performed using Stata (version 15.0) software. Interoceptive accuracy scores were calculated for each of the time intervals, and averaged to give an overall score. Mean heart rate (MHR) was assessed by calculating the participant’s heart rate across each time trial, and then averaging the data to give an overall MHR estimate.

The variables age and interoceptive sensibility (BPQ scores) were found to be nonnormally distributed and were transformed. The following variables were found to be nonnormally distributed and could not be transformed: BMI, EDE Global scores, HADS depression, interoceptive accuracy scores, and confidence in task performance scores. In addition to the nonnormal distribution, interoceptive accuracy scores were found to be highly skewed (skewness = −1.39, kurtosis = 5.32). Therefore, nonnormally distributed variables were analysed using nonparametric tests, and are summarized in the results using median and interquartile range (*IQR*) values instead of means and standard deviations (*SD*).

Group differences on each variable were calculated using *t*-tests, or Mann-Whitney U tests for nonparametric variables that could not be transformed. Correlations were performed within each group to establish relationships between the heartbeat tracking task, and the BPQ and confidence ratings.

Within the AN group only, a multiple linear regression analysis was performed with interoceptive accuracy (overall mean score) as the dependent variable to explore the relative contributions of autistic traits and alexithymia, while also controlling for the role of anxiety and depression as recommended by previous research (21). Correlational analyses were performed to assess relationships between confidence scores and clinical variables in the AN group.

## Results

### Clinical and Demographic Characteristics

In the AN group (*n* = 37), 31 participants had restrictive AN (83.78%), while 6 participants had binge/purge AN (16.22%). Mean illness duration was 9.41 years (*SD* 7.72). Twenty-nine participants were receiving treatment for their AN at the time of study participation (78.38%), and eight participants were not receiving treatment (21.62%). Of the participants receiving treatment, the majority (*n* = 23, 62.16%) were receiving outpatient treatment, and a minority (*n* = 6, 16.22%) were in inpatient treatment. Twenty-four participants with AN (64.86%) were taking psychotropic medication. Three participants with AN reported a prior diagnosis of autism. In addition, the majority (*n* = 24, 64.86%) of participants in the AN group reported at least one comorbid clinical diagnosis. The most common clinical diagnoses were depression (*n* = 15) and anxiety (*n* = 10), and *n* = 5 participants reported a diagnosis of borderline personality disorder. Diagnoses reported by only one participant were bipolar disorder, obsessive-compulsive disorder, and posttraumatic stress disorder.

Group differences are summarized in **Table 1**. Participants were matched on age and gender, and exhibited no differences in MHR. As expected, participants with AN had lower mean BMIs compared to the HC group, and scored higher on measures of alexithymia, ED symptomatology, autistic traits, depression, and anxiety.

**Table 1:** Clinical and demographic group characteristics.

	HC mean (*SD*) (*n* = 37)	AN mean (*SD*) (*n* = 37)	Test statistic	*p*	Effect size (*d*)
**Age (years)**	26.05 (7.13)	26.08 (8.05)	*t*(72) = -0.36	0.720	0.08
**Gender**	*n* = 35 female (94.59%), *n* = 2 male (5.41%)	*n* = 35 female (94.59%), *n* = 2 male (5.41%)		1.00	
BMI*	22.8 (4.4)	15.8 (1.2)	*U* = 0	<0.001	3.37
**Mean Heart Rate (MHR; beats per minute)**	72.27 (10.12)	69.19 (11.22)	*t*(72) = 1.24	0.219	0.29
**Alexithymia (TAS)**	41.76 (13.45)	61.43 (13.12)	*t*(72) = -6.37	<0.001	1.48
EDE-Q Global*	0.61 (0.89)	4.22 (1.33)	*U* = 10	<0.001	3.20
**AQ**	12.57 (6.80)	23.30 (10.36)	*t*(72) = -5.27	<0.001	1.22
HADS Depression*	2 (3)	9 (5) *n* = 36	134.5	<0.001	1.89
**HADS Anxiety**	6.08 (3.90)	13.17 (4.21) *n* = 36	*t*(71) = -7.46	<0.001	1.75

*Data nonnormally distributed. Medians and interquartile ranges presented, and data analysed using nonparametric methods.

### Interoceptive Accuracy

Heartbeat tracking scores are summarized in **Table 2**. There were no significant differences between groups on the overall heartbeat tracking score, or at any time point, with small effect sizes.

**Table 2:** Group differences in interoceptive accuracy scores.

	HC mean (*SD*) (*n* = 37)	AN mean (*SD*) (*n* = 37)	Test statistic	*p*	Effect size (*d*)
Interoceptive Accuracy*	0.67 (0.35)	0.74 (0.28)	*U* = 580.5	0.261	0.26
25 seconds*	0.68 (0.45)	0.83 (0.35)	*U* = 507	0.055	0.46
35 seconds*	0.71 (0.58)	0.75 (0.29)	*U* = 600	0.361	0.21
45 seconds*	0.70 (0.34)	0.71 (0.38)	*U* = 667.5	0.854	0.04
100 seconds*	0.68 (0.30)	0.77 (0.29)	*U* = 586.5	0.289	0.25

*Data nonnormally distributed. Medians and interquartile ranges presented.

### Interoceptive Sensibility

There were no significant differences between groups in interoceptive sensibility as measured by the BPQ (HC mean= 117.61 (*n* = 36, *SD* = 43.00), AN mean= 115.43 (*n* = 37, *SD* = 24.49), *t*(71 )= 0.21, *p* = 0.833, *d* = 0.05). The AN group did score significantly lower on their confidence rating in their interoceptive accuracy task performance, with a medium effect size (HC median = 50, *IQR* = 43.00, AN median = 40, *IQR* = 38), *U* = 477.5, *p* = 0.025, *d* = 0.54).

### Metacognitive Insight

In the HC group, there was no relationship between the heartbeat tracking task and BPQ scores (*r* = 0.09, *p* = 0.605). There was a significant positive correlation between heartbeat tracking scores and confidence ratings (*r* = 0.60, *p* <0.001). By contrast, in the AN group there was no correlation between the heartbeat tracking task and the BPQ (*r* = 0.17, *p* = 0.322), or the confidence ratings (*r* = 0.26, *p* = 0.117).

### Relationship With Clinical Variables

The relative contribution of autistic traits, alexithymia, anxiety, and depression to interoceptive accuracy were calculated using a regression analysis within the AN group only. There were no significant relationships between any of these clinical variables and interoceptive accuracy (**Table 3**).

**Table 3:** Relative contribution of clinical variables to interoceptive accuracy within the anorexia nervosa (AN) group only.

	*B*	*t*	*p*
**Autistic traits (AQ)**	0.00	0.02	0.987
**Alexithymia (TAS)**	0.05	0.20	0.846
**Anxiety (HADS)**	−0.21	−1.10	0.278
**Depression (HADS)**	−0.07	−0.34	0.736

Correlations between clinical variables and task confidence ratings were also explored within the AN group only. There were no significant relationships between confidence ratings and alexithymia, autistic traits, anxiety, or depression in the AN group. However, there was a significant negative relationship between confidence ratings and ED severity as measured by the EDE-Q Global score (*r* = −0.41, *p* = 0.012).

## Discussion

The overall aim of this study was to explore whether interoceptive accuracy as measured by the heartbeat tracking task is associated with alexithymia and autistic traits in AN. Contrary to the hypothesis that people with AN would exhibit lowered cardiac interoceptive accuracy compared to HC, the study found no significant differences between groups in heartbeat tracking performance. This is in line with a number of recent studies, including two that were published after the hypotheses for the current study were generated (12–14). The findings of the present study, and more recent research, contrast with previous research using the heartbeat tracking task in AN which found lowered accuracy in this population (10, 11). One potential explanation for this variation in findings are differences in the AN samples used in each study, such as differences in BMI, age, comorbidities, illness duration, and treatment status. For example, the participants with AN in this study were receiving a range of different treatments (inpatient, outpatient, or no treatment), compared to participants receiving self-help only in the Pollatos et al. (11) study. The participants in the current study additionally had lower BMIs, higher mean illness duration, and were slightly older compared to this initial study. This reflects concerns that heartbeat tracking task performance is associated with state-dependent factors (25). For example, Richard et al. (14) found that interoceptive accuracy was associated with inpatient treatment progress, with higher accuracy associated with higher BMIs and longer time in treatment (14).

The second hypothesis of this study was that people with AN would exhibit lowered interoceptive sensibility (self-perceived interoceptive aptitude) compared to HC. Findings on interoceptive sensibility were mixed: there were no differences between groups on the BPQ, a measure of self-reported awareness of bodily symptoms, but people with AN did report lower confidence in their interoceptive task performance. The third hypothesis of this study was that people with AN would exhibit poorer metacognitive insight, operationalised as group correlations between performance and BPQ/confidence ratings. The finding that there was a positive correlation between task performance and confidence ratings in the HC group, but not the AN group, suggests that people with AN may lack insight into their interoceptive abilities (55). Significantly, lower confidence ratings were correlated with higher ED symptomatology in the AN group, indicating that this lack of insight may be related to ED severity. If individuals with AN have less confidence in their ability to detect interoceptive sensations, this could result in a reliance on other cues, such as prior beliefs around likely interoceptive responses. The possibility that people with AN rely on predicted sensations, as opposed to the detection of actual sensations, is supported by research suggesting that people with AN find it difficult to detect actual interoceptive responses from anticipated responses (55, 56). Individuals with AN were more likely to falsely endorse changes in interoceptive sensation in the absence of stimulation, and reported more intense cardiorespiratory sensations compared to HC, during pre-meal states. This prediction error between actual and anticipated responses is also thought to be altered in other conditions with heightened prevalence in AN, including autism, anxiety and depression (31, 57). Future research should consider further investigating the concept of metacognitive insight in interoception in AN, in particular the role that this might play in interoceptive prediction errors.

Alternatively, the lower confidence ratings found in this study may reflect the fact that low self-esteem is very common in people with AN (58). Therefore, the findings of this study could reflect a generalised lack of confidence in ability, rather than a lack of confidence specific to interoceptive performance. It should be noted that the current results contrast with the findings of Lutz et al. (13) who found no difference between groups in task confidence ratings (13).

Finally, the study hypothesised that there would be an association between interoceptive accuracy, alexithymia and autism within the AN group. The findings of this study did not support this hypothesis, with no relationships found. Consequently, it is possible that interoceptive accuracy is not linked to alexithymia and autistic traits in AN, and is rather associated with other drivers, such as treatment duration or BMI (14). However, it should be noted that interoceptive accuracy, autism, and alexithymia have not consistently been linked in previous studies: two recent studies have found no associations between autism and interoceptive accuracy in adults (33, 34). Similarly, two additional studies have found no association between interoceptive accuracy and alexithymia (34, 59).

It is likely that these mixed findings on the relationship between cardiac interoceptive accuracy, alexithymia, and autism, and indeed for the inconsistent results surrounding interoceptive accuracy in AN, is related to the heartbeat tracking task itself. The heartbeat tracking task was chosen for the current study as it has been used in the majority of previous research on interoceptive accuracy in AN, alexithymia, and autism. However, as previously outlined, heartbeat perception can be influenced by a number of factors beyond the control of the current study. For example, a recent study exploring alexithymia and interoceptive accuracy in a sample of 287 participants initially found no relationship, and subsequently only detected a relationship after accounting for 10 additional control variables (47). Some of these variables were accounted for in group comparisons in the present study: for example, there were no significant differences between groups on age or MHR. However, groups in the current study significantly differed on other variables, including anxiety, depression, BMI, and alexithymia. Although the present study is the one of the largest studies on interoceptive accuracy in AN to date [*n*= 74 compared to *n* = 76 in Richard et al. (14)], it was not possible to account for the potential roles of all these variables owing to the relatively small sample size limiting the ability to perform a large multivariable regression analysis. Additionally, the nonparametric distribution limited the ability to control for variables in group comparisons using ANCOVAs. Finally, the current study did not include a control task to account for the possible influence of participant beliefs about heart rates, or the possibility that they were counting time rather than heartbeats. However, a strength of the current study was that it controlled for anxiety and depression while exploring the relationship between interoceptive accuracy, alexithymia and autism in people with AN (47).

Therefore, the findings of this study should be understood in the context of the limitation that there are a number of problems associated with using the heartbeat tracking task as a measure of interoceptive accuracy. Future research in this area should consider adapting the heartbeat task to control for potential covariates identified by Murphy et al. (47), or by modifying the task instructions to instruct participants to specifically count their felt heartbeats, rather than reporting an estimate (23). Alternatively, studies on interoception in AN could move away entirely from the heartbeat tracking task to a more robust measure of interoceptive accuracy. For example, recent studies on cardiac interoception in AN have instead used bolus intravenous infusions of isoproterenol to artificially raise heartbeat and respiratory rate in a controlled manner, and then asked participants to rate their changing sensations using a dial (55, 56). While this type of methodology is more invasive compared to the heartbeat tracking task, it does allow for a more highly controlled approach.

Significantly, these studies similarly found no difference in interoceptive accuracy, but did find prediction errors made specifically in the context of meal anticipation. This appeared to be related to heightened anxiety, and atypical interoceptive representation of the heartbeat: individuals with AN located sensations in the left side of their chest in the absence of actual stimulation (56). Further research should consider exploring aspects of interoception in AN other than accuracy, including the ability to discriminate between sensations, or magnitude estimations. The finding in these studies that altered interoception is potentially specific to meal anticipation also warrants further research. In the current study, proximity of the task to meals was not considered. It is possible that task performance, particularly for AN participants, could have been influenced by task timing in relation to meal anticipation. Interestingly, in the current study people with AN did not self-report generalised problems with detecting bodily symptoms, as measured by the BPQ. Taken together with previous research suggesting elevated difficulties as measured using the EDI (16), these findings support the possibility in AN are specifically associated with hunger and satiety sensations, or sensations associated with emotion detection only, rather than representing a generalised difficulty. Future research could consider focusing on whether interoceptive differences in AN are associated with specific states, such as heightened emotional arousal, hunger and satiety, or meal anticipation.

In conclusion, the current findings indicate that there are no differences in heartbeat tracking task performance in people with AN compared to HC, and that this performance is not associated with alexithymia or autistic traits within AN populations. However, these findings are presented in the context of potentially significant limitations with the chosen methodology. The study did find that people with AN potentially exhibit lower metacognitive insight. Recommendations are made for future research in this area.

## Data Availability Statement

The datasets for this article are not publicly available because the authors do not have permission to share the participant data publicly. Data is available upon request from Principal investigator of the study KT, kate.tchanturia@kcl.ac.uk.

## Ethics Statement

The studies involving human participants were reviewed and approved by North East - Newcastle & North Tyneside 2 Research Ethics Committee (18/NE/0193). The patients/participants provided their written informed consent to participate in this study.

## Author Contributions

All authors contributed to the design of the study. EK carried out data collection, and wrote the first manuscript draft. KT and CS contributed to the final manuscript. All authors read and approved the final manuscript.

## Funding

EK was supported by a Medical Research Council Doctoral Training Partnership studentship (MR/N013700/1). KT would like to acknowledge support from MRC and MRF Child and Young Adult Mental Health (MR/R004595/1) and support from the Health foundation, an independent charity committed to bring better health care for people in the UK (Ref: AIMS ID): 1115447.

## Conflict of Interest

The authors declare that the research was conducted in the absence of any commercial or financial relationships that could be construed as a potential conflict of interest.
